# Dropout in Online Education: A Longitudinal Multilevel Analysis of Elementary Students’ Extracurricular English Course Engagement in China

**DOI:** 10.3390/bs15040483

**Published:** 2025-04-07

**Authors:** Haotian Tan, Xueting Li

**Affiliations:** Department of Psychology, Renmin University of China, Beijing 100872, China; t.haotian@ruc.edu.cn

**Keywords:** online elementary education, class-level dropout, hierarchical linear model, cliff effect

## Abstract

While high dropout rates hinder the success of online education, little is known about the patterns of dropout beyond adult education, particularly regarding time-related variables. This study aims to address this gap by analyzing data from 219 online extracurricular English courses, involving approximately 300,000 Chinese elementary students. We employed a multilevel modeling technique and found that the dropout rate increased throughout the semester and peaked at chapter transitions. Furthermore, we also found that the dropout rate varied with grades and semesters. Younger students and those in the second semester were more likely to drop out from online education and exhibited a higher dropout rate during chapter transitions. These findings highlight the temporal dynamics of dropout in elementary online education in China and the variations across grade and semester, providing valuable implications for educators in designing more effective online learning environments. Future studies should further explore the generalizability of these patterns in other educational and cultural contexts.

## 1. Introduction

The emergence of distance learning technologies has established online education as a fundamental aspect of modern educational practices. Online education is generally defined as “the education form being delivered in an online environment through the use of the internet for teaching and learning” (Singh & Thurman, 2019). By removing the temporal and spatial constraints of traditional face-to-face instruction (Panigrahi et al., 2018), online education—particularly in the format of massive open online courses (MOOCs)—has witnessed rapid growth in universities (Perna et al., 2014). The COVID-19 pandemic further accelerated this transition, compelling educational institutions for all age groups to adopt online learning on an unprecedented scale (Lockee, 2021; Van Lancker & Parolin, 2020).

Despite its advantages, online education presents significant challenges, particularly high dropout rates, which negatively impact both students and educational institutions (Bağrıacık Yılmaz & Karataş, 2022; Perna et al., 2014). These challenges are evident in both adult online education (Bağrıacık Yılmaz & Karataş, 2022; Xing & Du, 2019; Perna et al., 2014) and K-12 (kindergarten through twelfth grade) online learning (Black et al., 2022). Consequently, understanding the factors that contribute to student dropout in online education has become a critical area of research (Abbott-Chapman et al., 2014; Borrella et al., 2022; Corry et al., 2017).

However, most studies are limited to adult education. While online K-12 education has been booming in recent years, there are few studies examining how elementary and secondary school students are learning online (Guggemos et al., 2022; Zheng et al., 2020). Given the substantial differences between adults and children in areas such as meta-cognitive abilities, self-control, learning habits, motivation, and social–family relations, the dropout patterns may differ (Broadbent & Poon, 2015; Koutsakas et al., 2020; Yan et al., 2021). As a result, the investigation of online K-12 education can provide new and valuable insights into student dropout behaviors. Furthermore, previous research has predominantly focused on educational practices within Western contexts, with limited exploration of non-Western cultural environments, particularly in China.

To address this gap, this study adopted a hierarchical linear model (HLM) to assess the dropout patterns based on data from K-12 online cram education in China. From a longitudinal multilevel perspective, we aimed to provide critical insights into how class-level dropout trajectories develop over the course of the semester. Additionally, we explored how factors such as grade and semester moderate these trajectories, shedding light on key variables that could inform more effective strategies for reducing the dropout rates in online education. The findings have important implications for improving student retention and engagement in K-12 online education.

### 1.1. Factors Behind the Dropout of Online Education

Unlike the situation in offline education, certain learner characteristics and learning strategies (e.g., self-regulation skills) are required of students to succeed in online education (de Barba et al., 2020; Hew et al., 2020), which has been proven challenging for some students, such as early-school-year students (Yan et al., 2021).

Researchers have identified various key antecedents of adult online education engagement, particularly in MOOCs, including internal factors such as personal traits and external factors like learning environment, course design, etc. (Akpen et al., 2024; Kocsis & Molnár, 2024; Lee & Choi, 2011; Rahmani et al., 2024; Salas-Pilco et al., 2022; Willging & Johnson, 2009). In recent years, with the development of socioformation theory, which posits that student engagement in education results from multiple factors, including the roles of parents and teachers, as well as technological and emotional aspects (Guerra-Macías & Tobón, 2025), studies have begun to focus on more complex educational interaction contexts. For example, using information from MOOC video data, Suen and Hung (2024) revealed the impact of teacher expressions and verbal appeal on emotional engagement in online education through deep learning algorithms. Haerawan et al. (2024) found that students engaged in online learning through interactive video had a 30% increase in viewing time, leading to a 25% improvement in learning outcomes.

Similar factors influence K-12 online learning, too. For example, Kurt et al. (2021) conducted a thematic analysis of interviews with Turkish students and teachers, highlighting the effects of policies, learning environment (e.g., access to electronic devices), personal characteristics (e.g., academic motivation, self-regulation), and instructional guidance. A study of 93 rural online schools distributed in 29 U.S. states discussed several main reasons for high school students dropping out, including scheduling and time constraints, lack of academic rigor and motivation, technology problems, difficulties with the online medium, lack of teacher immediacy, and parental influences (De la Varre et al., 2014). A cross-sectional study of over a thousand students in China also found that students’ Big Five personality traits, family involvement, and school support contributed to their engagement in online learning (P. Wang et al., 2023). These studies emphasize the complex factors influencing academic engagement across cultures and the need for collaboration between teachers, students, governments, and institutions to promote sustainable education (Tobon & Lozano-Salmorán, 2024).

### 1.2. The Time-Related Factors of Online Education Dropout and Its Fluctuations

Although cross-sectional studies have yielded substantial contributions to the field of education, we cannot overlook the fact that learning is an ongoing process that evolves over time (Getman et al., 2024). The studies relying on cross-sectional data struggle to capture the dynamic fluctuations in student engagement. A key feature of online education is its flexibility, allowing students to leave the virtual classroom at any moment, resulting in highly irregular learning trajectories (Broadbent & Poon, 2015; Maldonado-Mahauad et al., 2018). In long-term courses, students may drop out at any point and reengage later. It is crucial to conceptualize dropout as a dynamic process and apply longitudinal data analysis to better model the complex trajectories that students take in their educational experiences (Ameri et al., 2016).

Although research on the dynamic trajectories of online learning is still in its early stages, some studies suggest that the number of dropout students increases throughout the semester. For example, Wild and Heuling (2020), in their study on students in German cooperative higher education, documented a developmental progression of student dropout from 2016 to 2019 and observed a monthly increase in dropout risk. Through a predictive model using a deep learning algorithm, Xing et al. (2019) also found that students were more likely to drop out of online courses over time. Similarly, Greene et al. (2015) and Xie (2019) identified a rising trend in dropout rates within higher education settings.

Beyond the long-term evolution of learning processes, student dropout is also influenced by short-term circumstances (Willett & Singer, 1991), with dropout rates fluctuating at specific stages. For instance, Chen et al. (2020) identified a “cliff effect”, where dropout rates overshoot at chapter transitions. Moreover, later transitions in a course sequence led to higher dropout rates—a pattern validated in two popular MOOCs. Similarly, Muir et al. (2019) examined semester-long fluctuations in student engagement at an Australian university, highlighting time-sensitive factors such as workload management, instructor presence, and competing demands beyond the academic ones. These findings suggest that dropout is a dynamic and context-dependent process, shaped by both instructional structures and external pressures. Therefore, a longitudinal research methodology is needed to capture the temporal patterns and underlying factors driving the dropout trends.

However, limited longitudinal studies focused on the dynamic trajectories of student dropout beyond the adult context. One study investigated student engagement in online education for German students in grades 4 to 10 and found that, within one year after enrollment, the proportion of student engagement declined at an accelerating rate over time (Spitzer et al., 2021). This finding is consistent with research on adult online education. But the generalizability of these conclusions to non-Western contexts remains insufficiently explored. Furthermore, it remains unclear whether elementary online education exhibits the cliff effect, underscoring the need for further investigation in this area.

### 1.3. The Particularity of Elementary Online Education in China

In this study, we mainly focus on one significant format of K-12 online education: extracurricular education, also known as shadow education, which is provided by private institutions and companies through cram schools that charge certain fees (Bray, 2013). These education programs operate outside of formal schooling, supplement regular education, and assist students in enhancing their academic performance and preparing for exams (Luo & Chan, 2022; Yung & Bray, 2021). Cram schools initially emerged in many Asian countries, such as China, Japan, and Korea, where academic competition is fierce at the elementary and high school levels (Chung, 2013). Thus, extracurricular education students attend them mainly for exam preparation, while higher education learners focus on skill expansion (Kwok, 2004).

In fact, with the development of China’s economy and society, online elementary education, primarily in the form of cram schools, has become a new cultural trend in Chinese society. A large number of students are required to complete their school tasks while also engaging in additional live streaming learning during their spare time, which places tremendous pressure and burden on both students and parents (Bao, 2024; Zhang et al., 2024). Unlike MOOCs, elementary online education requires younger students with more self-regulation skills (Yan et al., 2021). According to the cyclical model of self-regulated learning (Zimmerman & Moylan, 2009), students typically navigate three stages in their learning process: forethought, performance, and self-reflection. However, factors such as low self-control, poor time management, and weak academic self-efficacy can disrupt this cycle, increasing the likelihood of dropout behaviors.

Evidence suggests that students in the early school years often have underdeveloped metacognitive skills, which hinder their ability to effectively self-regulate. This includes challenges in managing their time and sustaining effort in online learning environments. (de Barba et al., 2020; Guggemos et al., 2022; Lee et al., 2013). Labrador et al. (2019) found that individual factors, such as age, influence the student dropout rates throughout a course, with older students demonstrating greater persistence. Considering the cognitive development characteristics of younger students, it is reasonable to expect that elementary online education also exhibits a similar trend of increasing dropout rates over time. Moreover, online extracurricular education is designed to help students cope with school tasks. Therefore, Chinese elementary students may prioritize formal schoolwork over extra online lessons. However, during chapter transitions, additional academic tests are conducted in the tutoring sessions to help students prepare for exams, which in turn increases their pressure. As a result, during this period, students with lower self-regulation abilities may struggle to balance school tasks and extracurricular tutoring. This difficulty in multitasking may increase their likelihood of dropout (Kizilcec & Halawa, 2015). Thus, we expected that the cliff effect may be observed in online cram schools for Chinese primary students.

### 1.4. The Impact of Grades and Semesters in Student Dropouts

The dropout rates may be influenced by individual characteristics and course design (Lee & Choi, 2011; Rahmani et al., 2024). Therefore, this study further examined the role of grade and semester sequence in the dropout rate and its “cliff effect” in online cram schools for Chinese primary school students. Given that these characteristics are universal across different types of education, the exploration of their impact can provide valuable insights for broader educational practices.

The relationship between grade level and dropout rates in online tutoring requires further exploration. Research on offline education suggests that the risk of dropout increases with the grade level, potentially due to heavier academic workloads or greater financial pressures. For example, De Laet et al. (2015) found that academic engagement among students in Grades 4 to 6 decreased with age. Similarly, a large longitudinal study in Australia observed a general decline in engagement among students aged 6 to 9 (Martin et al., 2024), a trend also reported for students aged 7 to 12 (Burns et al., 2019).

However, online tutoring differs from traditional educational settings, and whether it follows the same pattern remains an open question. On the one hand, it may depend on offline education, where higher-grade students face greater academic pressure and may adjust their study schedules based on long-term academic planning, which leads them to opt out of online tutoring. On the other hand, the flexibility of online tutoring demands greater self-regulation and time management skills (Xavier & Meneses, 2022), which lower-grade level students may struggle with (Geng et al., 2024), increasing their likelihood of dropout. Considering that both of these perspectives are possible, this study aims to further clarify the issue by introducing the factor of grades.

The impact of semester order on dropout rates remains unclear, as existing research has yielded inconsistent findings. Some studies suggest that the risk of dropout is highest in the first semester and gradually decreases over time (e.g., Kemper et al., 2020). This pattern may stem from students’ initial struggles in adapting to a new learning environment; once they become familiar with the course structure and expectations, their likelihood of persistence increases. Additionally, financial commitments, such as tuition fees and sunk costs, may further discourage dropout in later semesters (Heublein, 2014). Conversely, other studies indicate that the dropout rates tend to rise as the semester progresses (e.g., Pascarella & Terenzini, 1980; Geisler et al., 2023). This may be due to students reassessing their academic goals after earning a certain number of credits or facing external pressures such as financial constraints and family responsibilities. Moreover, parents often enroll their children in online tutoring programs to supplement their learning and improve their academic performance in offline classes. As a result, students may struggle to balance mastering the material from online courses with their regular school workload. Given these conflicting findings, this study aimed to analyze the dropout trends across different semesters to provide a clearer understanding of the role of semester order on the dynamic dropout process.

### 1.5. The Current Study

**Figure 1:**
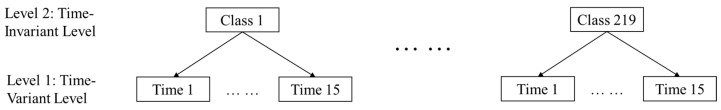
Data structure for the research analysis. The time-invariant level indicates that the independent variable did not change over time at this level, while the time-variant level indicates that the independent variable changed over time at this level.

Research on online education has mainly focused on adult-oriented MOOCs and cross-sectional data, leaving the dropout rates among elementary students largely unexplored. The moderating impact of factors such as grade and semester on dropout patterns remains unclear. This study used data from 219 English tutoring classes, each enrolling students in a 15-week live-streaming online course, to explore student dropout trajectories in elementary online education in China (see data structure in Figure 1), providing key insights for understanding online education in Chinese elementary schools and filling a gap in this relevant field. Specifically, our study addressed the following four research questions (RQ):

**RQ1:** *How do dropout rates fluctuate over the course of a program at the class level?*
**RQ2:** *Does elementary online education exhibit the cliff effect, and does this effect persist in subsequent chapters?*
**RQ3:** *How do student grade, semester, and their interaction moderate the dropout trajectories?*
**RQ4:** *How do student grade, semester, and their interaction moderate the cliff effect?*

For research questions 1 and 2, we propose the following hypotheses:

**H1.**  *As the online course progresses, the dropout rates will increase steadily.*

**H2.**  *Students in cram schools are more likely to drop out during chapter transitions, with this trend becoming more pronounced as the course progresses.*

It is important to note that, due to the scarcity of research on the moderating impact of grade and semester on online educational trajectories, we did not make explicit hypotheses for research questions 3 and 4. Therefore, we conducted exploratory analyses.

Given the spatiotemporal nested structure of the data in our study (with different lessons nested within different classes; see Figure 1) and the core aim of exploring the dropout rate trajectories, we employed an HLM for analysis. HLMs have been widely used in online education research (Lin et al., 2023), are well suited for nested data structures, and allow us to observe temporal trends (Bryk & Raudenbush, 1987). We did not use survival analysis because our outcome variable, the class-level average dropout rate, is continuous. Survival analysis, on the other hand, requires time-to-event data with a binary 0–1 variable to indicate the occurrence of an event at a specific time. Therefore, survival analysis was not applicable in this context. A more detail comparison can be seen in Stryhn and Christensen (2014). In this study, our HLM included time-variant factors at level 1 to assess the dropout trends and the cliff effect. Level 2 incorporated time-invariant factors, like grade and semester, to examine their moderating impact on the student dropout patterns.

## 2. Materials and Methods

### 2.1. Participants and Data Acquisition

In this study, the data were collected by a company in China that specializes in online education for elementary and middle school students. During the fall semester of 2021 (the first semester) or the spring semester of 2022 (the second semester), the company offered extracurricular online English courses to elementary students for a fee. The courses were delivered via live streaming during non-school hours, with each 90 min session held once a week for a total of 15 sessions per semester. The course content was aligned with the curriculum taught in schools.

Each semester’s course contained three chapters: Chapter 1 had 4 or 5 lessons, Chapter 2 had 4 lessons, and Chapter 3 had 7 lessons. There were four chapter transitions (T4 or T5, T8, and T15), which provided opportunities to observe the hypothesized cliff effect. The students were encouraged to actively engage in live online sessions and interact with their teachers. Furthermore, the students were required to pass a chapter-transition test before progressing to new content after completing each chapter.

Data collection was conducted by the online education company. Before participation, the students and their parents were informed that their course engagement data would be recorded. The data were recorded only after the participants signed an informed consent form. Additionally, to strictly adhere to ethical standards, we refrained from obtaining personal data information of the students from the data provider. The students’ online course engagement was averaged at the class level and recorded as the class dropout rate.

The initial dataset contained 247 classes. Any classes with incomplete data for all 15 sessions were excluded from the analysis. After cleaning, valid data from 219 classes covering 3285 sessions and nearly 300,000 students nationwide were retained. All students were from grades 1 to 6 and were categorized by the platform into three groups: lower (grades 1–2), middle (grades 3–4), and higher (grades 5–6). There were 40 classes in the lower-grade group, 69 in the middle-grade group, and 110 in the higher-grade group.

### 2.2. Variables

The dependent variable in this study was the class-level student dropout rate, calculated as the ratio of the number of dropouts to the total number of students. The courses were delivered through synchronous live streaming. Therefore, if a student failed to attend a session or dropped out during the session, they were considered to have dropped out. Only students who completed the full 90 min session were considered to have engaged.

The independent variables in this study were divided into two categories: time-variant predictors and time-invariant predictors. At the time-variant level, our model focused on two key predictors: the number of lessons (${time}_{ti}$) and the cliff effect (${cliff}_{ti}$). The cliff effect was coded as 1 if a lesson included the chapter-transition test and as 0 otherwise. Additionally, we included an interaction effect between time and the cliff effect (${time\times cliff}_{ti}$). A significant interaction effect would indicate that the cliff effect changed over the duration of the course.

At the time-invariant level, our models incorporated two important predictors: the grade year (${grade}_{i}$) and the semester (${semester}_{i}$). The grade year was assessed across three levels (1 = lower grade, i.e., grades 1–2; 2 = middle grade, i.e., grades 3–4; 3 = higher grade, i.e., grades 5–6), while the semester was categorized into two levels (0 = first semester, 1 = second semester). Additionally, we included an interaction effect between grade year and semester (${semester\times grade}_{i}$) to examine any potential combined effects between these variables.

### 2.3. Data Analytic Strategies

We employed the HLM to analyze the longitudinal and multilevel data, using the R 4.20 lmerTest package (version 3.1-3). The effects of time-variant level (level 1), time-invariant level (level 2), and cross-level interactions were examined simultaneously. To enhance interpretability and mitigate issues related to multicollinearity (Hofmann & Gavin, 1998), we performed grand-mean centering on all variables, except for the categorical variables (i.e., the cliff effect, grade year, and semester). We focused on theory validation rather than on group heterogeneity, which led us to construct random-intercept and fixed-slope models (Bhutoria & Aljabri, 2022). Four models were considered. Firstly, a null model was estimated, which did not include any level 1 or level 2 variables. Next, the level 1 variables were inputted in model 2. Then, the level 2 variables were entered into the model 3, and finally the cross-level variables were added in model 4.

To assess the model fit, we employed the Akaike information criterion (AIC) and Bayesian information criterion (BIC) fit indices, which are commonly used to compare the relative fit of different models (Akaike, 1974; Schwarz, 1978). Additionally, we calculated two types of *R*^2^ (marginal and conditional *R*^2^) to estimate the effect size of the models. The marginal *R*^2^ represents the variance explained by the fixed factors, while the conditional *R*^2^ represents the variance explained by both the fixed and the random factors. The mixed model was constructed as follows:

Level 1 model:

(1) $$Y_{ti}=\pi_{0i}+\pi_{1i}\times {time}_{ti}+\pi_{2i}\times {cliff}_{ti}+\pi_{3i}\times {time\times cliff}_{ti}+e_{ti}$$

where $Y_{ti}$ denotes the dropout rates for class *i* at lesson *t*, and the intercept $\pi_{0i}$ and slope $\pi_{1i}$, $\pi_{2i}$, $\pi_{3i}$ are time-invariant-level coefficients.

The level 2 model had these coefficients as responses:

(2) $$\pi_{0i}=\beta_{00}+\beta_{01}\times {grade}_{i}+\beta_{02}\times {semester}_{i}+\beta_{03}\times {semester\times grade}_{i}+\gamma_{0i\;}$$

(3) $$\pi_{1i}=\beta_{10}+\beta_{11}\times {grade}_{i}+\beta_{12}\times {semester}_{i}+\beta_{13}\times {semester\times grade}_{i}$$

(4) $$\pi_{2i}=\beta_{20}+\beta_{21}\times {grade}_{i}+\beta_{22}\times {semester}_{i}+\beta_{23}\times {semester\times grade}_{i\;}$$

(5) $$\pi_{3i}=\beta_{30}+\beta_{31}\times {grade}_{i}+\beta_{32}\times {semester}_{i}+\beta_{33}\times {semester\times grade}_{i\;}$$

where $\;{time}_{ti}$, ${cliff}_{ti}$, ${time\times cliff}_{ti}\;$ denote level 1 predictors, ${grade}_{i}$, ${semester}_{i}$, ${semester\times grade}_{i}$ denote level 2 independent variables, $e_{ti}$ denotes normally distributed level 1 residuals with mean zero and level 1 variance θ, and $\gamma_{0i}$ denotes level 2 residuals following a normal distribution with mean zero and level 2 variance ψ.

Additionally, to minimize the potential influence of researcher bias, anonymized data and the data analysis methods were provided to an expert, who was completely unaware of our study and hypotheses, for independent analysis. The results from this independent analysis are consistent with our findings.

## 3. Results

### 3.1. Fluctuations in Elementary Student Dropout over Time

To examine the fluctuations in elementary student dropout throughout the semester, HLMs with restricted maximum likelihood estimation were employed. The analysis began with a random intercept-only unconditional model (Model 1), in which no variables were specified. The intraclass correlation coefficient (ICC) was 0.242, indicating that 24.2% of the variance in student dropout was attributable to within-class fluctuations over time. This result underscored the necessity of a multilevel linear approach to appropriately model both within- and between-class effects (Bryk & Raudenbush, 1987).

Next, multilevel models with random intercepts were used to examine both linear and quadratic trajectories of student dropout. The linear time model included mean-centered discrete time, the cliff effect, and their interactions as predictors, while the quadratic time model further incorporated the squared term of discrete time. The results indicated that the linear effect was significant (*b* = 0.51, *p* < 0.001), whereas the quadratic effect was not (*b* = 0.04, *p* > 0.05). Given these findings, the linear model was selected (Model 2). As shown in Table 1, the estimated average dropout rate was 9.04%, with a weekly increase of 0.51% thereafter. This result is consistent with hypothesis 1, which suggests that, as the course progresses, there is a cumulative time effect on student dropout in elementary online education.

**Figure 2:**
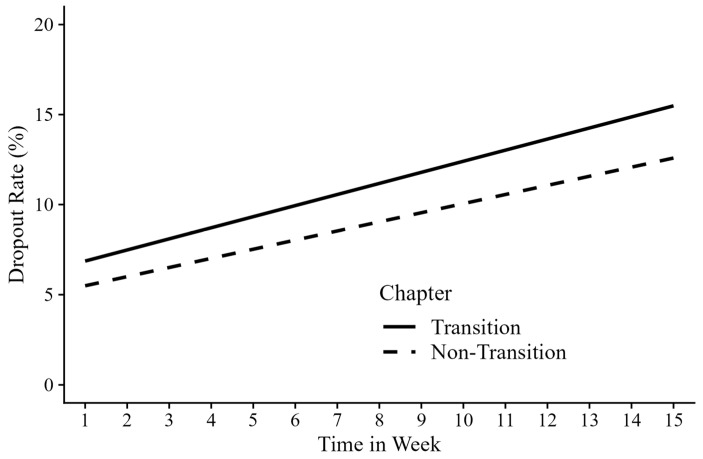
The simple slope plot of the interaction between time and the cliff effect.

The cliff effect was found to be significantly and positively associated with student dropout (*b* = 2.14, *p* < 0.001), suggesting that the dropout rates were higher at chapter transitions (11.17% ± 4.71%) than at non-transitions (9.04% ± 3.42%). Further analyses examined the interaction between cliff and time, revealing a significant positive interaction effect (*b* = 0.11, *p* < 0.001). This finding suggests that the risk of dropout at chapter transitions increased progressively over time (see in Figure 2). Simple effect analyses showed that the estimated slope for dropout was 0.62% per week at chapter transitions and 0.51% per week at non-chapter transitions (*p*s < 0.001).

**Figure 3:**
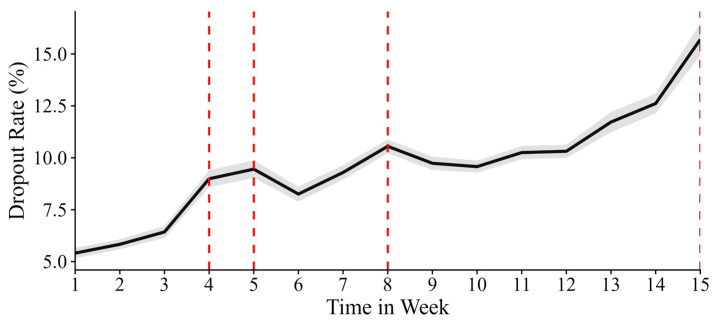
Plotting rate of dropout (%) at each week, taking cliff into consideration and controlling other covariates at their means. The grey area represents the 95% confidence interval. The red dashed line indicates the times with chapter transition.

Furthermore, to better understand the magnitude of the cliff effect over time, we estimated the probability of dropping out at key time points, controlling for all other covariates. As illustrated in Figure 3, the dropout rate, which increased steadily over time, exhibited sharp spikes at chapter transitions. Notably, these spikes became more pronounced as the course progressed, with the dropout rates estimated at 8.99% at T4, 9.45% at T5, 10.55% at T8, and reaching 15.70% at T15. These results support hypothesis 2, indicating that a significant cliff effect is present in elementary online education and intensifies over time.

### 3.2. Moderating Effects of Grade and Semester on Dropout Fluctuations over Time

To examine the potential influence of grade and semester on student dropout (RQ3 and RQ4), these variables along with their interactions with time-related variables (time and cliff), were incorporated into the multilevel model. As shown in Model 3, grade was significantly and negatively associated with the probability of dropout (*b* = −1.10, *p* < 0.001), indicating that students in higher grades were less likely to drop out ($M_{lower}\;$= 10.92% ± 3.75%, $M_{middle}\;$= 9.53% ± 3.58%, $M_{higher}$ = 9.18% ± 4.08%). However, semester was not significantly associated with dropout (*b* = −0.53, *p* > 0.05), even though the dropout rate was numerically higher in the second semester (10.20% ± 5.44%) than in the first (9.33% ± 2.91%). These findings suggest a protective effect of grade on dropout in online education among elementary school students, as well as a potential influence of semester.

In Model 4, we introduced the interaction between time-variant and time-invariant factors to further explore the moderating impact of grade and semester on fluctuations in dropout rates. The four-way interaction between class variables (grade and semester) and time-related variables (time and cliff) was not significant (*b* = 0.05, *p* > 0.05). The three-way interaction between time, cliff, and grade was also not significant (*b* = 0.02, *p* > 0.05).

**Figure 4:**
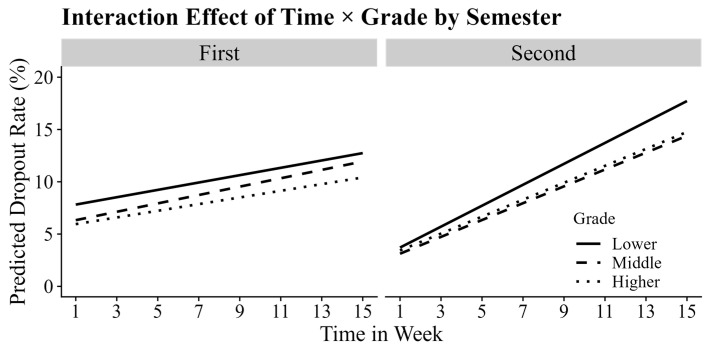
The simple slope plot of the three-way interaction between time, grade, and semester. The left subplot, labeled “First”, represents the simple slopes of time × grade for the first semester, while the right subplot, labeled “Second”, represents the simple slopes of time × grade for the second semester. Different lines represent different grades.

The three-way interaction between grade, semester, and time was statistically significant (*b* = −0.04, *p* = 0.05), indicating that the pattern of dropout over time varied across grades and semesters. As illustrated in Figure 4, simple slope analyses revealed that in the first semester, the rate of increase in dropout rates did not significantly differ across grades (*p*s > 0.05). In contrast, during the second semester, the rate of increase in dropout rates was significantly higher in the lower grades than in the middle (*p* < 0.001) and higher grades (*p* < 0.001), while no significant difference was observed between middle and higher grades (*p* > 0.05). Specifically, the weekly dropout rate increased by 1.11% in the lower grades, 0.96% in the middle grades, and 0.96% in the higher grades. Additionally, the average rate of increase in dropout during the second semester (1.11%) was significantly higher than that in the first semester (0.31%), *b* = −0.8, *p* < 0.001, indicating a steeper rise in student dropout as the academic year progressed.

**Figure 5:**
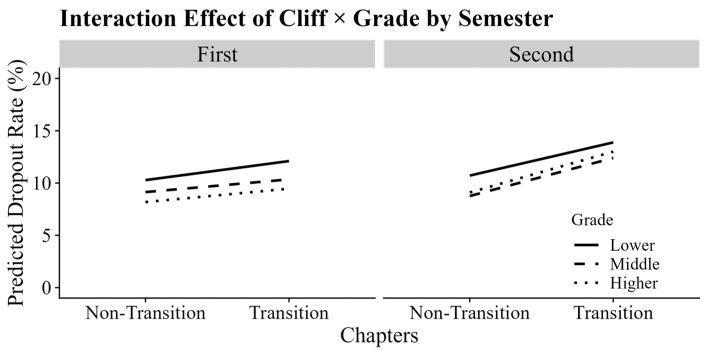
The simple slope plot of the three-way interaction between cliff, grade, and semester. The left subplot, labeled “First”, represents the simple slopes of cliff × grade for the first semester, while the right subplot, labeled “Second”, represents the simple slopes of cliff × grade for the second semester. Different lines represent different grades.

Further analyses revealed a significant three-way interaction between grade, semester, and cliff, which was positively associated with the dropout rate (*b* = 0.57, *p* < 0.01). As illustrated in Figure 5, in the first semester, the dropout rate at chapter transitions was 1.82% higher in the lower grades compared to that at non-chapter transitions, reflecting a larger cliff effect. In contrast, the cliff effects for the middle (1.22%) and higher grades (1.28%) were lower (*p*s < 0.05), with no significant difference between middle and higher grades (*b* = −0.06, *p* > 0.05). However, in the second semester, the overall cliff effect was higher than in the first semester (*p* < 0.05), which aligns with the grade-related trend in dropout rates. Nonetheless, the differences in cliff effects across the three grade groups were not significant (*p*s > 0.05), with cliff effects of 3.17% for the lower grades, 3.64% for the middle grades, and 3.91% for the higher grades.

**Figure 6:**
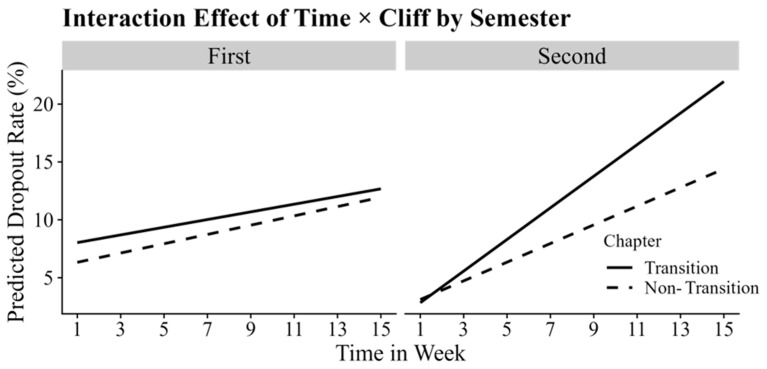
The simple slope plot of the three-way interaction between time, cliff and semester. The left subplot, labeled “First”, represents the simple slopes of time × cliff for the first semester, while the right subplot, labeled “Second”, represents the simple slopes of time × cliff for the second semester. Different lines represent different chapter types.

Additionally, a significant three-way interaction was found between time, cliff effect, and semester on dropout (*b* = 0.53, *p* < 0.001). Specifically, in the second semester, the weekly dropout rate at chapter transitions increased by 1.39%, compared to 0.87% at non-chapter transitions, which contributed to a growing cliff effect over time (*b* = 0.52, *p* < 0.001). This contrasts sharply with the relatively stable time variation of the cliff effect in the first semester (see in Figure 6, left panel). These findings suggest an increasing sensitivity to chapter transitions as the academic year progressed.

## 4. Discussion

Moving beyond prior research on dropout trajectories in online higher education, the present study observed unique dropout fluctuations in online cram schools for Chinese elementary students and offers novel insights into the dynamic patterns of dropout. A steady increase in class-level dropout rates was found throughout the academic year, with variations across different grades and semesters. A pronounced spike in dropout rates was observed at the end of each chapter—resembling a “cliff effect”—and this effect became more pronounced as the course progressed. These findings provide key implications for future research and teaching practices in China’s elementary school online education.

### 4.1. Dropout Patterns in Elementary Online Education

The study identified a steady increase in the class-level dropout rate among Chinese elementary school students during the COVID-19 pandemic. Such findings align with existing research on adult (Greene et al., 2015; Wild & Heuling, 2020; Xie, 2019; Xing et al., 2019) and K-12 education (Spitzer et al., 2021; F. Wang et al., 2024). Through dynamic tracking at the large-scale class level, this study expands the understanding of the changing trends in dropout rates among different developmental stages, revealing a general pattern of increasing dropout in online education.

Several factors may have contributed to this trend, including heightened anxiety and loneliness associated with the pandemic, as well as practical challenges such as insufficient learning space, screen fatigue, and various distractions (Fabian et al., 2022). Additionally, considering that our sample was from an English course, which is a second language learning context in China, it exhibited strong teacher-centered characteristics and limited teacher–student interactions (W. Wang & Gao, 2008). Students’ motivation for learning a second language also plays a crucial role in their participation in the learning process (You et al., 2016). In the context of learning English as a foreign language, learners’ metacognitive strategies may be less effective (Li, 2014). The specific nature of second language learning may also have an impact on the increase in dropout rates.

A more critical explanation, however, may be the cumulative effect of the dropouts. As the semester progressed, the dropout behavior became self-reinforcing, leading to fewer students remaining engaged. This implies that most students who dropped out did not reengage again. In fact, Chen and Kizilcec (2020) found that only 36.3% of students who dropped out for a week re-engaged within two weeks. Similarly, Saqr and López-Pernas (2021) reported that about 49% of low-engagement university students remained absent for the rest of the course. From the perspective of the self-regulated learning theory (Zimmerman & Moylan, 2009), learners are expected to actively manage their learning process by setting goals, monitoring progress, adjusting strategies, and maintaining motivation. However, when students drop out, their established learning plans and routines are disrupted. Additionally, the accumulation of unfinished tasks can create a “pile-up effect”, increasing cognitive and emotional pressure and initiating a negative feedback loop. Consequently, students may adopt avoidance coping strategies instead of actively regulating their learning behavior.

Taken together, these findings suggest that online education should prioritize the early identification of students struggling with adaptation and timely support them (Bañeres et al., 2023). Equally important is developing strategies to help short-term dropouts regain their self-regulation skills and reduce the psychological and behavioral barriers to re-engagement (Dignath et al., 2008), thereby preventing the further escalation of dropout rates.

### 4.2. The Moderating Effect of Grade and Semester on Dropout in Elementary Online Education

We also found that this overall increase in dropout rate was moderated by grade and semester. In general, regardless of the semester, grade appeared to act as a protective factor, slowing the increase in dropout rates as students progressed to higher grades. The increase in dropout rates tended to slow as students grew older. However, when we examined the differences between the two semesters, we found that this effect was only present in the second semester. In the first semester, the dropout rates fluctuated without a clear trend.

This finding contrasts with studies on offline education engagement, which emphasize the increasing dropout rates as grade levels go up (Burns et al., 2019; De Laet et al., 2015; Martin et al., 2024). However, a study on the developmental trajectories of offline education in China partially aligns with our observation, showing that student engagement increased consistently from grade 3 through the first semester of grade 5, but began to decline in the second semester (Zhu et al., 2019). The differences between online and offline education, as well as the varying cultural contexts, further emphasize the need for more in-depth and comprehensive research.

Such divergence between online and offline education may highlight the distinct impact of course structure on students’ sustained engagement. Offline classes typically offer more opportunities for face-to-face interactions with teachers and peers, which are often lacking in online education for students. These elements are particularly beneficial for younger students, who often require external guidance to maintain focus and enthusiasm (Geng et al., 2024; Yan et al., 2021). This aligns with Martin et al. (2024), who found that teacher support is crucial for maintaining engagement and motivation. This is especially important in second language learning, yet it is often overlooked by English teachers in China (W. Wang & Gao, 2008; You et al., 2016). In addition, online courses demand greater autonomy and time management skills (Xavier & Meneses, 2022). Younger students, who struggle with self-regulation (Geng et al., 2024), are more likely to drop out without external guidance, especially in the Chinese extracurricular tutoring programs that impose immense academic pressure on students (Yan et al., 2021). As a result, grades may play a protective role in online education dropout by enhancing academic autonomy and cognitive ability. For students in lower grades, their online academic engagement is more likely to deteriorate over time.

The differences during the semester further emphasize the importance of students’ cognitive development and the online education environment. In the Chinese elementary education environment, the second semester content is a review and further extension of the first semester’s material. This means that students need to utilize their learning skills and strategies more effectively to tackle more difficult academic tasks. Therefore, students in lower grades face more severe challenges in online education during the second semester compared to students in upper grades. In contrast, the first semester is the start of learning tasks. To help students better adjust to the learning process, teachers provide more support and assistance, and the tasks are relatively easier. As a result, differences across grades are less noticeable in the first semester.

These findings echo socioformation theory, which emphasizes that student engagement is shaped not only by individual motivation and abilities but also by educational structures and sociocultural contexts (Guerra-Macías & Tobón, 2025). The unique characteristics of online education make students more vulnerable to external factors such as family background, self-discipline, and social support (Rahmani et al., 2024). Therefore, incorporating contextual teaching strategies that provide structured support may be crucial for reducing the dropout rates in online education settings.

### 4.3. The Cliff Effect in Elementary School Online Education

In elementary school online education, we replicated the cliff effect observed in higher education (Chen et al., 2020). Specifically, the dropout rates spiked during chapter transitions, with the effect becoming more pronounced as the course progressed. Cross-level analysis showed that in the second semester, each additional online English class had an increased probability of dropout during chapter transitions of 0.59%, which contrasts sharply with the relatively stable cliff effect observed in the first semester. According to self-determination theory, the satisfaction of students’ intrinsic motivation is crucial for their participation in online education (Sun et al., 2019). In fact, throughout the semester, students’ learning motivation generally declines with time (Martin et al., 2024; Darby et al., 2013). The trend of increasing cliff effects over time may be related to the continuous decline in students’ intrinsic motivation (Chen et al., 2020). Cram classes in China usually have a highly repetitive learning schedule, which may cause students to feel bored at the end of the chapter, reducing their intrinsic motivation and making them choose to drop out of the course. Specifically, in China’s elementary education system, the second semester serves as an extension of the content from the first semester. Students often experience heightened academic pressure in the second semester, which may further amplify this time-sensitive dropout pattern, leading to a higher rate of cliff effect growth in the second semester.

One of the most interesting findings in our study is how the cliff effect itself changed across student grades and semesters. In the first semester, the cliff effect was less pronounced in higher- (1.28%) and middle-grade (1.22%) courses compared to lower-grade courses (1.86%). However, in the second semester, there was no significant difference in cliff effect across the three grade levels and the average cliff effect was higher (over 3%). This finding is similar to the changes in dropout rates across grades and semesters, influenced by a combination of students’ intrinsic motivation, cognitive development characteristics, and the specific features of online education practices in China. In students’ first semester, as we described earlier, teachers may provide extra support to help students adjust. During the phase of transitioning between semesters, teachers need to do more preparation and design tests, which reduces the external support for students. Therefore, for lower-grade students, it may be more difficult to adapt to the short-term changes in teacher support, which leads to a decline in their intrinsic motivation and an increase in the dropout levels (Fryer & Bovee, 2016; Guvenc, 2015), ultimately causing a larger cliff effect. In contrast, in the second semester, overall, less teacher support and heavier academic tasks may lead to a higher cliff effect across grades.

These findings offer teachers some instructional guidance, helping them know when and whom to focus on in different semesters. Therefore, in order to reduce the increase in dropout rates during chapter transitions (i.e., the cliff effect), the platform design can consider introducing staggered assessments and pre-transition motivational modules (Luburić et al., 2021). Specifically, through decentralized quizzes and gamified challenges, students can be provided with immediate feedback at each stage of learning, helping them maintain their interest and pace in learning and preventing motivational gaps after completing units (Looyestyn et al., 2017). However, it needs to be emphasized again that, given that the study of online education for students is still in its early stages, our understanding of online education engagement is still limited, especially in the unique context of online education in English in China. Any interpretation and intervention suggestions should be treated with caution to avoid over-generalizing the conclusions to other cultures or education systems in the absence of more empirical support.

### 4.4. Limitations and Future Directions

There are several limitations of the present study that should be acknowledged.

First, because the data came directly from an online education company, commercial and ethical limitations constrained the range of data that we could access. As a result, we could only analyze the dropout rates at the class level and were unable to systematically explore factors such as individual-level data (e.g., gender, age, learning experiences, academic stress, socioeconomic status, parental involvement), which thus prevented an examination of individual differences. As mentioned earlier, individual factors and institutional factors play a crucial role in shaping students’ experiences in online education (Greenhow et al., 2022). Factors such as the socioeconomic status can also act as barriers to access online education, particularly the paid online shadow education. Future research should build upon our foundation to further investigate how these factors influence the trajectory of students’ engagement in online education and validate relevant theoretical explanations, ultimately supporting the development of more student-centered and personalized teaching practices.

Second, while the large-scale data somewhat mitigated sampling errors and helped reduce researcher bias, the unique model of an elementary online education in English course in China limits the generalizability of the findings. The differing educational models between Eastern and Western contexts may shape students’ engagement patterns in online education. Future research could further explore the similarities and differences across different cultures and courses, leading to more culturally specific teaching reforms.

Third, the data used in this study were obtained from a private company during the 2021–2022 period, when China was still experiencing the effects of COVID-19. Consequently, the possibility of sampling bias cannot be fully ruled out. Although the COVID-19 pandemic accelerated the development of online education, it is undeniable that it could have had a potential impact on students’ academic engagement. Therefore, the results of our study may also have been influenced by the pandemic. However, given the consistency of our findings with other studies, we believe that the results remain convincing. Future research could further explore the differences in student engagement in online education before and after the pandemic to clarify this issue, thereby promoting the development of education in the post-COVID-19 era.

Finally, education in the digital era should focus more on the sustainability of future education and its impact on students (Abo-Khalil, 2024), an aspect that cannot be simply summarized through quantitative analysis. The utilization of qualitative research methods, such as focus group interviews, will be essential to explore subjective causes (e.g., motivation loss, parental pressure). By combining qualitative and quantitative methods, we will achieve a more comprehensive understanding of the factors behind student dropout in online education.

## 5. Conclusions

Our study is one of the first studies to apply multilevel modeling to analyze temporal dropout patterns (e.g., cliff effect) in elementary online education, combining grade-, semester-, and chapter-level dynamics. These findings yield important insights for future research on elementary school online education as well as for the design and management of online education courses. First, students may be more likely to drop out of the learning environment during transitions between chapters. Accordingly, course design and teaching strategies should prioritize supporting students during these periods to facilitate a smoother adaptation to the changing content. Second, the differences between grades and learning stages appeared to be particularly pronounced in elementary online education, with younger students showing a more noticeable tendency to drop out. Online education professionals should pay more attention to the specific needs of younger students and provide grade-specific support. Finally, student dropout appeared inevitable and continuously increasing. Thus, building real-time engagement monitoring systems is essential for detecting at-risk students and providing timely interventions. Educators should prioritize supporting these students to help them reengage and continue their learning. Overall, this study provides a template for analyzing dropout dynamics in diverse educational contexts, offering valuable implications for educators in designing more effective online learning environments and urging cross-cultural collaborations to build equitable online learning ecosystems.

## Figures and Tables

**Table 1:** Multilevel models predicting student dropout trends over the semester.

Variables	Model 1	Model 2	Model 3	Model 4
Fixed effects				
Intercept (SE)	9.61 *** (0.14)	9.04 *** (0.15)	11.20 *** (0.49)	11.27 *** (0.49)
*Level 1*				
time		0.51 *** (0.01)	0.51 *** (0.01)	0.41 ***(0.03)
cliff		2.14 *** (0.09)	2.14 *** (0.09)	1.88 *** (0.23)
time × cliff		0.11 *** (0.02)	0.11 *** (0.02)	−0.13 * (0.05)
*Level 2*				
grade			−1.10 *** (0.21)	−1.04 *** (0.21)
semester			−0.53 (1.01)	−0.80 (1.02)
grade × semester			0.69 (0.40)	0.54 (0.40)
*Cross-level interaction*				
time × grade				−0.02 * (0.01)
time × semester				0.59 *** (0.06)
time × semester × grade				−0.04 * (0.02)
cliff × grade				−0.23 * (0.10)
cliff × semester				1.02 * (0.47)
cliff × semester × grade				0.57 ** (0.19)
time × cliff × semester				0.53 *** (0.11)
time × cliff × grade				0.02 (0.02)
time × cliff × semester × grade				0.05 (0.04)
Random parameters				
$\sigma_{e}^{2}$	11.67	4.94	4.94	2.47
$\sigma_{u0}^{2}$	3.71	4.16	3.48	3.64
ICC	24.12%	45.71%	41.33%	59.57%
Model fit				
Deviance	17,778.80	15,141.40	13,845.30	12,976.10
AIC	17,784.80	15,153.40	13,865.30	13,012.10
BIC	17,803.00	15,190.00	13,925.60	13,121.90
Marginal $R^{2}$	0	0.41	0.45	0.60
Conditional $R^{2}$	0.24	0.68	0.68	0.84

Notes: The dependent variable in all models was the class-level dropout rate, expressed as a percentage. Time was a course time variable, ranging from 1 to 15, with units in weeks. Cliff represented whether the course included a chapter-transition test, with a value of 1 if the test was included. Grade was categorized into three levels: lower, middle, and higher, encoded as 0, 1, and 2, respectively. Semester had two levels, with the first semester encoded as 0, and the second semester encoded as 1. All regression coefficients were unstandardized, with the estimated standard errors provided in parentheses. ICC = intraclass correlation coefficient; AIC = Akaike information criterion; BIC = Bayesian information criterion; *R*^2^ means the proportion of variance explained for each model. Level 1, N = 3285. Level 2, N = 219. *** *p* < 0.001, ** *p* < 0.01, * *p* < 0.05.

## Data Availability

The data presented in this study are available on request from the corresponding author. The data are not publicly available due to the requirement of protecting privacy.

## Abbreviations

The following abbreviations are used in this manuscript:

MOOCs	Massive open online courses
K-12	Kindergarten through twelfth grade
HLM	Hierarchical linear model
